# Targeting Immune Checkpoint Molecules to Eliminate Latent HIV

**DOI:** 10.3389/fimmu.2018.02339

**Published:** 2018-10-15

**Authors:** Zoe Boyer, Sarah Palmer

**Affiliations:** ^1^Centre for Virus Research, Westmead Institute for Medical Research, Sydney, NSW, Australia; ^2^Faculty of Medicine and Health, University of Sydney, Sydney, NSW, Australia

**Keywords:** memory T cells, T cell exhaustion, immune checkpoint molecules, PD-1, CTLA-4, latent HIV reservoir

## Abstract

The advent of antiretroviral therapy (ART) has seen a dramatic decrease in the morbidity and mortality of individuals infected with human immunodeficiency virus (HIV). However, ART is not curative and HIV persists in treated individuals within a pool of infected CD4^+^ memory T cells. The targeting and elimination of these cells, termed the latent HIV reservoir, may be essential in establishing a cure for HIV. Current HIV reservoir research is focused on identifying cells that harbor latent, replication-competent, HIV provirus using specific cell surface markers. Recently, studies have turned to immune checkpoint (IC) molecules, such as programmed cell death protein 1 (PD-1). IC molecules are regulators of the immune system and have previously been linked to HIV infection. Furthermore, cells isolated from treated individuals co-expressing PD-1 alongside other IC molecules are enriched for HIV DNA. Administration of a IC blocking antibodies resulted in an increase of cell-associated HIV RNA within an individual, indicating the potential for this therapeutic to be utilized as a latency reversing agent. IC inhibitors could target CD4^+^ T cells expressing IC molecules and possibly enhance HIV transcription, allowing for the elimination of these cells by either ART or the immune system. However, treatment with IC inhibitors has been associated with toxicities such as immune-related adverse events and therefore future studies should proceed with caution.

## Introduction

Current HIV reservoir research is focused on targeting and eliminating latently infected cells to establish a functional cure for HIV. To achieve this, researchers are utilizing cell surface markers to identify which cell types harbor greater quantities of HIV proviral DNA. Due to the error-prone nature of reverse transcription, HIV DNA often contains mutations that render the provirus incapable of producing new infectious virions (1–3). Only proviruses that contain all replication-essential genes are considered genetically intact and are replication competent (2, 4). It is these genetically-intact proviruses, harbored by memory CD4^+^ T cells, that are responsible for the viral rebound seen following the interruption of antiretroviral therapy (ART) and thus must be targeted (5). Studies have shown that levels of these latently infected cells, termed the functional HIV reservoir, are established early during acute infection and remain stable (6, 7), with little decay occurring after more than 7 years of therapy (4, 8–10). Moreover, due to the long half-life of memory cells that harbor latent HIV, as well as ongoing cellular proliferation (10, 11), it is estimated that more than 60 years of continuous therapy would be necessary to eliminate the virus from an individual (12).

The traditional memory CD4^+^ T cell subsets include central memory, transitional memory, and effector memory (T_CM_, T_TM_, and T_EM_, respectively). Initial investigations into memory cell subsets revealed T_CM_ cells to be enriched for replication-competent HIV provirus (13, 14). However, studies were limited by either design or the methods employed in their investigation. The study performed by Chomont et al. (13) identified T_CM_ as being enriched for inducible HIV DNA and showed that the contribution of T_EM_ cells was significantly less. However, T_EM_ are more difficult to isolate as they comprise a smaller proportion of total memory CD4^+^ T cells compared with their counterparts. In fact, in this study, T_CM_ cells made up more than 45% of total CD4^+^ T counts whilst only 10% was attributed to T_EM_ cells. Therefore, researchers were unable to identify significant amounts of HIV DNA within the T_EM_ cell population. The latter study, performed by Soriano-Sarabia et al. failed to include the T_EM_ cell subset in its investigation preventing a full assessment of all memory T cell subsets. With the advent of next-generation sequencing (NGS), we are able to sequence HIV DNA and gain insight into the location of genetically-intact and presumably replication-competent provirus, and consequently assess the contributions of cell populations to the functioning HIV reservoir (3, 15, 16).

Data obtained using NGS revealed T_EM_ cells to contain increased proportions of genetically-intact HIV provirus (3) when compared with other memory cell subsets, indicating that T_EM_ cells contribute significantly to the functioning latent reservoir. Preliminary data investigating the role of activation status of a cell and the latent HIV reservoir has indicated that the presence of the classical activation marker HLA-DR corresponds to an increased proportion of genetically intact integrated HIV provirus (17). Despite these findings, whether genetically-intact provirus isolated from cells expressing HLA-DR is capable of replicating remains to be demonstrated. Nevertheless, this concept supports the data implicating T_EM_ cells as defining the replication-competent reservoir, as the T_EM_ phenotype is one of increased cellular activation. It has become clear that cells can be found in a partially activated state and that the concept of cellular activation is better represented by a sliding scale than an on/off switch (18).

Investigations into HLA-DR as a marker of the latent reservoir have only begun in recent years. Prior to data published linking the marker with cells harboring integrated, genetically-intact HIV DNA (17, 19), HLA-DR was thought to indicate cells that were transcriptionally active and as such producing new infectious viral progeny—thus not in a latent state. The fact that cells expressing the activation marker HLA-DR are still capable of harboring latent HIV provirus has encouraged the exploration of other known markers of cellular activation. One such marker is the programmed cell-death protein 1 (PD-1), a known marker of immune regulation that is also categorized as an activation marker. Despite the inhibitory signaling it instigates within the cells which express it, PD-1 has been shown to cluster with HLA-DR (20) and is classified as a late stage cellular activation marker. The remainder of this review will discuss the evidence supporting the expression of PD-1, and other immune markers, as being intrinsically linked to latently infected cells and how this marker could be exploited in future HIV therapeutic approaches.

## Role of immune checkpoint molecules

In response to chronic viral infections, the immune system attenuates the effector function of T cells in a process known as T cell exhaustion. It is hypothesized that this process evolved to protect against the tissue-damaging consequences of a prolonged immune response, characteristic of ongoing chronic viral infections (21). T cell exhaustion was initially identified as specific to CD8^+^ T cells, however studies have demonstrated CD4^+^ T cells are also prone to exhaustion (22). It is worth noting that although T cell exhaustion can lead to the death of T cells, effector-function of exhausted cells can also be rescued (23). Exhausted T cells are characterized by expression of various inhibitory receptors, termed immune checkpoint (IC) molecules, such as PD-1 and cytotoxic T-lymphocyte-associated antigen 4 (CTLA-4). The ligation of these IC molecules induces intracellular signaling that inhibits cytokine production and ultimately results in cellular apoptosis (21). Current immunotherapies manipulate IC molecules in the treatment of several cancers, including melanoma, renal cancer and non-small cell lung cancer (NSCLC). Antibodies directed at IC molecules block their inhibitory effects, preventing the exhaustion of CD8^+^ T cells and enhancing anti-tumor immunity (24). The exhausted immune state that occurs in response to tumor formation is analogous to that which is seen during chronic infection with a virus—for example, HIV (21). This has sparked a wave of interest in IC molecules and their role in HIV infection and latency.

The expression of PD-1 and CTLA-4 on CD4^+^ T cells in the context of both treated and untreated HIV infection has been explored in several studies. In untreated infection, HIV-specific CD8^+^ T cells upregulate PD-1 expression as their effector function declines. PD-1 is also upregulated on HIV-specific CD4^+^ T cells, with *in vitro* PD-1 blockade restoring the proliferative nature of these cells that is lost following exhaustion (25). Moreover, PD-1 expression, in conjunction with other exhaustion markers TIM-3 and LAG3, was found to predict both time to viral rebound (>400 viral copies/ml) in patients that underwent treatment interruption (20) and disease pathogenesis in patients not receiving active treatment (26). With regards to CTLA-4, expression of this molecule by HIV-specific CD4^+^ T cells is elevated in all classes of infected individuals, with *in vitro* blockade resulting in significant levels of proliferation of these cells (27).

Recently, associations between IC molecules and the HIV latent reservoir have been identified. Specifically, memory CD4^+^ T cells (T_CM_ and T_TM_) expressing higher levels of PD-1 were found to harbor larger quantities of HIV DNA when compared to their PD-1 low counterparts (13). However, this study failed to investigate the replication competency of the integrated proviruses isolated, meaning the contribution of these cells to the latent reservoir is yet to be fully elucidated. A more recent study demonstrated that CD4^+^ T cells, taken from treated individuals, co-expressing IC molecules PD-1, TIGIT, and LAG3 contain 10-fold the amount of integrated HIV DNA when compared with IC molecule negative CD4^+^ T cells. Moreover, CD4^+^ T cells expressing any one of these markers alone were found to harbor replication-competent integrated HIV DNA (28). This same study identified that cells expressing PD-1 were enriched for HIV genomes exclusively in T_EM_ and T_TM_ cells, supporting the findings of Hiener et al. (3). Additionally, CD4^+^ T cells expressing both PD-1 and CXCR5—markers of T_FH_ cells—isolated from lymph nodes of long-term supressed individuals have also shown to harbor replication-competent HIV provirus (29). CTLA-4 has also been linked with the HIV latent reservoir, with CTLA-4^+^ CD4^+^ T cells (T regulatory cells) isolated from the lymph nodes of treated macaques shown to be enriched for SIV DNA (30).

Cells expressing IC molecules that harbor HIV provirus could not only comprise the latent reservoir but also contribute to its maintenance. The genetic characterisation of integrated HIV DNA revealed a large portion is genetically defective and identical (10, 11, 13), indicating ongoing cellular proliferation maintains the persistent HIV reservoir. It is known that PD-1 is a marker of both homeostatic and antigen-induced cellular proliferation (31, 32). Moreover, a preliminary study which sequenced HIV DNA isolated from PD-1 expressing cells found a large expansion of identical sequences, some of which were genetically intact (preliminary data from our laboratory, data not shown). These findings signify that natural cellular mechanisms could potentially contribute to the maintenance and persistence of the replication-competent HIV within PD-1 cells and represent a barrier in achieving a full cure for HIV.

It is possible that expression of PD-1 is correlated with the formation of the latent HIV reservoir. HIV infected cells expressing PD-1 will have attenuated gene expression and will not produce viral proteins that contribute to viral cytopathic death of the cell. Instead these PD-1^+^ cells will survive, with the provirus integrated into their cellular genome (33). Further research is required to fully elucidate the mechanistic role PD-1 expression may play regarding the establishment and/or the perpetuation of HIV latency within memory CD4^+^ T cells. There has been some suggestion that PD-1 expression is indicative of the presence of transcription factors that may inhibit HIV-1 transcription. One such factor is Blimp-1, which has been shown to be associated with exhausted CD4^+^ T cells in the context of chronic infection (34). This same study reported that exhausted CD4^+^ T cells correlated with increased expression of PD-1. Another study found Blimp-1 expression to be elevated within memory CD4^+^ T cells that contribute to the latent HIV reservoir (35). It is therefore conceivable that Blimp-1 and PD-1 expression may facilitate conserving latency in memory T cells.

Collectively, these data indicate that PD-1 is a clear marker of memory CD4^+^ T cells that harbor latent HIV provirus. However, the question remains as to what proportion of the latent reservoir is represented by cells expressing this marker and whether it is a feasible target in eliminating HIV infection. Already, one study has identified a positive correlation between the percentage of PD-1^+^ CD4^+^ T cells and the amount of integrated HIV DNA (PD-1^+^ CD4^+^ expression at 64% correlates with HIV DNA levels of ~1100 copies/10^6^ CD4^+^ T cells) (28). A preliminary study from our laboratory employing full-length sequencing of the integrated HIV genome, using samples obtained from supressed patients, identified PD-1^+^ CD4^+^ memory T cells harbor greater proportions of genetically intact provirus in contrast to PD-1^−^ equivalents (preliminary data from our laboratory, data not shown).

## Current latency reversing agents

**Figure 1:**
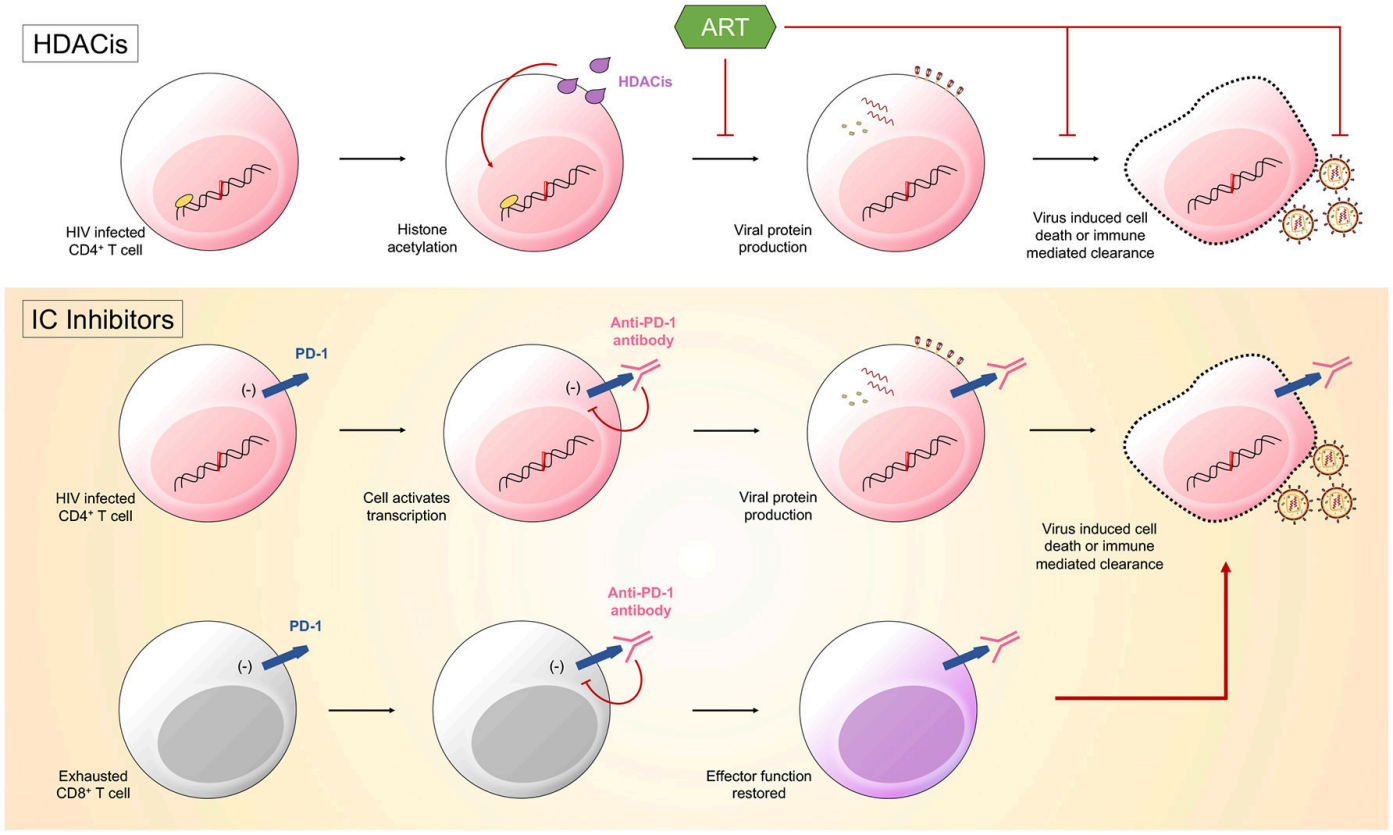
Latency reversing agents (LRA) are one half of the “shock and kill” approach. They function to induce HIV transcription. Histone deacetylase inhibitors (HDACis) promote histone acetylation and induce transcription of viral products. Immune checkpoint (IC) inhibitors could also function as LRA. Anti-PD-1 antibodies bind PD-1 on both HIV infected CD4^+^ T cells and HIV-specific CD8^+^ T cells. The binding of PD-1 by the antibody will disinhibit signals sent into the cells by the immune checkpoint molecule. Cells will become transcriptionally active, with CD4^+^ T cells producing viral products that ultimately result in the death of the cell, and CD8^+^ T cells having effector function restored, allowing them to target infected CD4^+^ T cells.

One approach to reduce the latent HIV reservoir is to combine current ART regimes with therapeutics that reactivate the latent form of the virus, a method known as “shock and kill.” The justification for this approach is that for the remaining hidden pool of virus to become visible to both the individual's immune cells and ART, it must be reactivated. This could be accomplished by treating individuals with latency reversing agents (LRA) that result in the transcription of latent, integrated HIV provirus. Various LRA exist, such as histone deacetylase inhibitors (HDACis) and protein kinase C class drugs, with studies having already proved their safety and the ability to increase levels of HIV transcription (36). These agents aim at directly modifying chromatin and reversing transcriptional latency and have been shown *in vitro* and *in vivo* to promote histone acetylation and increase HIV transcription, measured as cell-associated unspliced HIV RNA in resting memory CD4+ T cells (37) (Figure 1). Recent studies investigated the impact of the HDACis, vorinostat, panobinostat, and romidepsin upon the genetic composition of the HIV reservoir. HIV RNA sequences isolated from participant cells after therapy with these HDACis were found to be genetically diverse indicating that the drugs stimulated a broad activation of proviruses from latently infected cells (38, 39). However, multiple studies have shown that these drugs fail to significantly decrease the size of the latent reservoir (40). As we now know that some latently infected cells are already in a state of partial activation and express IC molecules (19, 28), it is conceivable that drugs could be used to target these cells via the expression of various markers, such as PD-1 and CTLA-4.

## Immune checkpoint inhibitors and HIV infection

There is now a push to optimize IC inhibitors currently used in the treatment of malignancies for the therapeutic treatment of HIV. Hypothetically, treatment would work in two potential ways: through enhancing HIV-specific CD8^+^ T cell effector function and through reversing HIV latency. It has already been established with SIV rhesus macaque animal models that the administration IC blockades have significant effects, with PD-1 blockade resulting in rapid expansion of SIV-specific CD8^+^ T cells with increased functionality (41) and CTLA-4 blockade resulting in increases in plasma viremia and T cell activation (42). IC blockades could function to reverse HIV latency through limiting inhibitory signals sent from IC molecules into cells harboring latent HIV. Preventing these inhibitory signals would result in increased gene expression and the subsequent production of viral proteins, causing these cells to become “visible” to both the immune system and ART, as well as susceptible to virus mediated cytotoxicity (33) (Figure 1).

Already there have been both case-reports and clinical trials that have investigated the use of IC blockades to treat malignancies in HIV-infected patients (Table 1). Two recent studies describe an HIV-infected individual who received treatment for melanoma in the form of multiple rounds of a CTLA-4 blocker (48) followed by the single administration of a PD-1 blocker (49). Following the administration of anti-CTLA-4 (ipilimumab), a 20-fold increase in cell-associated HIV RNA was measured in CD4^+^ T cells. After receiving the single dose of anti-PD-1 antibody (nivolumab), a 25-fold increase in cell-associated HIV RNA was observed (48, 49). These data indicate an increase in HIV transcription and a potential reversal of latency. Moreover, another case report of malignancy in an HIV-infected patient treated using PD-1 blockers demonstrated increased HIV transcription with a concurrent decrease in both reservoir size and PD-1^+^ CD4^+^ T cells after dosing (46). Conversely, a recent study analyzed data obtained from HIV-infected patients receiving treatment for NSCLC with a PD-1 blocker and came to different conclusions. Lavolé et al. (44) showed that both the proviral load and CD4^+^ T cell counts remained stable in patients receiving multiple doses of nivolumab, suggesting the drug does not function as an LRA as efficiently as previously thought (44). Furthermore, a second study found no consistent changes in either virological or immunological status in HIV-infected participants treated for malignancy with PD-1 blockers (45). However, it is worth noting that these studies all enrolled small cohorts (between 1 and 10 participants) and investigated doses that were optimized for the treatment of cancer. Only one study to date has explored IC inhibitors for the treatment of HIV in participants without any malignancies, using an anti-PD-L1 antibody. This study, a phase-I dose-escalation, observed increased Gag-specific CD8^+^ T cells in 2 of the 6 dosed participants. As this study was performed to assess the safety of the therapeutic, a low dose was used (0.3-mg/kg) and participants were monitored for adverse events (50). However, the investigation was halted due to observed retinal toxicities seen in concurrent macques studies. It is worth noting that IC inhibitors are associated with relative toxicities, with adverse events (AE) ranging from mild (diarrhea, fatigue, rash) to more severe (pancreatitis, nephritis, neurological conditions) (51). Future investigations should be cautious in their approach in regard to these AE.

**Table 1:** Summary of published trials and case-reports investigating immune checkpoint inhibitors in HIV.

**Paper**	**Model type/trial type**	**IC molecule targeted**	**Findings**	
			**Virological**	**Immunological**
(41)	SIV-infected rhesus macaques	PD-1 (*clone EH12-1540*)	Significant reduction in plasma viremia. Macaques treated during late chronic stage of infection had viral RNA copies drop below pre-treatment levels and delayed disease progression.	Rapid expansion of SIV-specific CD8^+^ T cells.
(42)	SIV-infected rhesus macaques	CTLA-4 (*MDX-010)*	Significant increase in plasma viremia.	Increased levels of T cell activation
(43)	Case report of HIV/HCV co-infected patient with malignancy (melanoma)	CTLA-4 (*ipilimumab)* then PD-1 *(pembromlizumab)*	Viral loads did not increase following administration of treatment.	No immune-related adverse events experienced.
(44)	HIV-infected participants with malignancy (NSCLC)	PD-1 (*nivolumab*)	Viral loads remained undetectable.	CD4^+^ T cell counts remained stable.
(45)	HIV-infected participants with malignancy	PD-1 (*nivolumab* or *pembromlizumab*)	No consistent changes in CD4^+^ T cell-associated HIV RNA or DNA or plasma viremia.	PD-1 binding decreased following initiation of therapy. No consistent changes in frequency of total or activated CD4^+^ or CD8^+^ T cells.
(46)	Case report of HIV-infected patient with malignancy (NSCLC)	PD-1 (*nivolumab)*	Transient increase in plasma HIV copies. Overall decrease in cell-associated HIV DNA.	Total CD4^+^ and CD8^+^ counts remained stable. Decrease in PD-1^+^ T cells.
(47)	Case report of HIV-infected patient with malignancy (NSCLC)	PD-1 (*nivolumab*)	Transient increase in cell-associated HIV DNA levels.	Transient increase in IL-6 levels. Transient increase in CD4^+^ and CD8^+^ T cell counts. Decrease of PD-1 expression by T cells.
(48)	Case report of HIV-infected patient with malignancy (melanoma)	CTLA-4 (*ipilimumab)*	Cyclical decrease in plasma HIV RNA levels following each dose of antibody, with an overall decline from 60 to 5 copies/ml. Cell-associated unspliced HIV RNA from CD4^+^ T cells increased ~20 fold.	Increase in total CD4^+^ T cell numbers.
(49)	Case report of HIV-infected patient [from Wightman et al.(48)] with malignancy (melanoma)	PD-1 (*nivolumab)*	Cell-associated unspliced HIV RNA increased ~25 fold. Ratio of cell-associated unspliced HIV RNA:HIV DNA significantly increased.	No changes reported.
(50)	Otherwise healthy HIV-infected participants	PD-L1 (*BMS-936559)*	No consistent changes in cell-associated RNA or DNA.	Significant increase in HIV-specific CD8^+^ T cells in 2 of 6 treated participants.

As CD4^+^ T cells that express both single and multiple IC molecules are enriched for HIV DNA (28), it is unlikely that an effective HIV-directed immunotherapy would involve only a single IC inhibitor. Rather, a combination of multiple blockers would be required for a synergistic approach to target all cells harboring latent HIV. The efficacy of combination immunotherapy has already been established in the treatment of NSCLC and other malignancies (52). However, the use of multiple IC inhibitors is associated with an increase in AE when compared with therapy using only one IC inhibitor (51). Furthermore, further research into exploiting other IC molecules, such as TIGIT and LAG-3, as targets for a potential HIV LRA is warranted, as cells expressing these markers contain integrated HIV DNA (28).

## Conclusion

Any curative strategy for HIV will need to eliminate the functioning latent reservoir. Previous approaches have used LRA, such as HDACis, to reawaken and eliminate cells latently infected with HIV. However these drugs do not decrease the size of the latent reservoir (40). As HIV latency research continues, new markers of latently infected cells are being identified (3, 17). IC molecules, such as PD-1, are promising therapeutic targets for the elimination of the HIV latent reservoir, as CD4^+^ T cells expressing IC molecules harbor latent, replication-competent HIV (13, 20, 28, 30). IC inhibitors are already used in a clinical setting and have transformed the treatment of cancer. Further investigation into these drugs, their effectiveness in treating HIV and the adverse immune events they could potentially cause is warranted. Furthermore, the impact of administering multiple classes of IC inhibitors should be investigated as it is unlikely that a single class will be sufficient to reactivate the HIV reservoir.

## Author contributions

Both ZB and SP made a significant and intellectual contribution to the work and approved it for publication.

### Conflict of interest statement

The authors declare that the research was conducted in the absence of any commercial or financial relationships that could be construed as a potential conflict of interest.
